# 4067 Cancer-related fatigue during combined treatment of androgen deprivation therapy and radiotherapy is associated with mitochondrial dysfunction

**DOI:** 10.1017/cts.2020.373

**Published:** 2020-07-29

**Authors:** Josephine K. Liwang, Li Rebekah Feng, Brian Wolff, Jeniece Regan, Sarah Alshawi, Leorey Saligan

**Affiliations:** 1 National Institutes of Health

## Abstract

OBJECTIVES/GOALS: Combined androgen deprivation therapy (ADT) and radiation therapy (RT) is the standard of care treatment for non-metastatic prostate cancer (NMPC). Despite the efficacy, treatment-related symptoms including fatigue greatly reduce the quality of life of cancer patients. The goal of the study is to examine the influence of combined ADT/RT on fatigue and understand its underlying mechanisms. METHODS/STUDY POPULATION: Sixty-four participants with NMPC were enrolled. Fatigue was assessed using the Functional Assessment of Cancer Therapy–Fatigue. Mitochondrial function parameters were measured as oxygen consumption from peripheral blood mononuclear cells (PBMCs) extracted from participants’ whole blood. An ADT/RT-induced fatigue mouse model was developed with fatigue measured as a reduction in voluntary wheel-running activity (VWRA) in 54 mice. Mitochondrial function was assessed in the ADT/RT mouse brains using Western blot analysis of Glucose transporter 4 (GLUT4) and transcription factor A, mitochondrial (TFAM). RESULTS/ANTICIPATED RESULTS: Fatigue in the ADT group was exacerbated during RT compared to the non-ADT group. This effect was specific to fatigue, as depressive symptoms were unaffected. PBMCs of fatigued subjects exhibited decreased ATP coupling efficiency compared to non-fatigued subjects, indicative of mitochondrial dysfunction. The ADT/RT mice demonstrated a synergistic effect of ADT and RT in decreasing VWRA. Brain tissues of ADT/RT mice exhibited decreased levels of GLUT4 and TFAM suggesting that impaired neuronal metabolic homeostasis may contribute to fatigue pathogenesis. DISCUSSION/SIGNIFICANCE OF IMPACT: These findings suggest that fatigue induced by ADT/RT may be attributable to mitochondrial dysfunction both peripherally and in the central nervous system (CNS). The synergistic effect of ADT/RT is behaviorally reproducible in a mouse model, and its mechanism may be related to bioenergetics in the CNS.

